# Cytotoxicity of Ferulic Acid on T24 Cell Line Differentiated by Different Microenvironments

**DOI:** 10.1155/2013/579859

**Published:** 2013-05-08

**Authors:** Chiung-Chi Peng, Charng-Cherng Chyau, Hui-Er Wang, Chi-Huang Chang, Kuan-Chou Chen, Kuang-Yu Chou, Robert Y. Peng

**Affiliations:** ^1^Graduate Institute of Clinical Medicine, College of Medicine, Taipei Medical University, 250 Wu-Hsing Street, Taipei 11031, Taiwan; ^2^Research Institute of Biotechnology, Hungkuang University, 34 Chung-Chie Road, Shalu County, Taichung 43302, Taiwan; ^3^Department of Food and Applied Biotechnology, Hungkuang University, 34 Chung-Chie Road, Shalu County, Taichung 43302, Taiwan; ^4^Department of Urology, School of Medicine, College of Medicine, Taipei Medical University, 250 Wu-Hsing Street, Taipei 11031, Taiwan; ^5^Division of Urology, Department of Surgery, Shin Kong Wu Ho-Su Memorial Hospital, 95 Wen Chang Road, Taipei 111, Taiwan

## Abstract

Ferulic acid (4-hydroxy-3-methoxycinnamic acid) (FA) is a ubiquitous health beneficial phenolic acid. Although FA has shown a diversity of biological activities including anti-inflammatory, antihypercholesterolemic and anticancer bioactivities, studies revealing its adverse effects are accumulating. Recently, 3D-cultures are shown to exhibit uniquely biological behaviors different from that of 2D cultures. To understand whether the cytotoxicity of FA against the T24 cell line (a bladder cancer cell line) in 2D-culture could consistently retain similar bioactivity if cultured in the 3D-systems, we conducted this experiment with 2 mM FA. Much higher cytotoxicity was found for 3D- than 2D-culture, showing (2D vs. 3D): apoptotic rates, 64% and 76%; cell killing rates, 3.00 × 10^5^ cells mmol^−1^·h^−1^ and 2.63 × 10^6^ cells mmol^−1^·h^−1^, attaining a 8.77-fold. FA upregulated the activities at 72 h (2D vs. 3D in folds that of control): SOD, 1.73-folds (*P* < 0.05) versus 3.18 folds (*P* < 0.001); and catalase, 2.58 versus 1.33-folds. Comparing to the control (without FA), Bcl-2 was prominently downregulated while Bax, caspase-3 and cleaved caspase-9 were more upregulated in 3D-cultures (*P* < 0.05). Conclusively, different microenvironments could elicit different biological significance which in part can be ascribed to different mass transport rate.

## 1. Introduction

Ferulic acid (4-hydroxy-3-methoxycinnamic acid) (FA), an effective component of many Chinese medicinal herbs like *Angelica sinensis, Cimicifuga heracleifolia,* and *Lignsticum chuangxiong*, is a ubiquitous phenolic acid in the plant kingdom [1]. FA exhibits many physiological functions including antioxidant, antimicrobial, anti-inflammatory, antithrombosis, antihypercholesterolemic, anticancer activities, and spermatozoa activating bioactivity [1]. Interest in the role of flavonoids to act as health benefits is emerging owing to their potential biological activities. However, to date, epidemiologic studies exploring the role of flavonoids in human health have been inconclusive [2]. Some studies supported the protective effect of flavonoids on cardiovascular disease and cancer, others demonstrated no effect [2], and interestingly a few suggested them to be potentially harmful [3]. More recently, we demonstrated FA to be nephrodamaging when used for a long-term treatment for chronic kidney disease (CKD) [4].

Conventional adherent tissue culture involves growing cells on solid flat surfaces as two-dimensional (2D) monolayers. Although such practices are routine and suitable for transformed or immortalized cell lines, dedifferentiation and loss of specialized functions occur when primary cells are removed from their host tissue and grown as 2D monolayers. This is generally believed to be a result of the dissociation of primary cells from their native three-dimensional structure *in vivo* to their two-dimensional propagation on flat impermeable substrates *in vitro* [5–7]. As such, there is a continuing need to develop tissue culture systems which can either promote redifferentiation of laboratory cell lines or prevent primary cell lines from dedifferentiating. 

The reason(s) eliciting different biological outcomes by different microenvironments is still unclear. With an aim to understand more about the cellular physiology and conversely the different cytotoxicity of a given flavonoid like FA that may occur in different microenvironments as specified by the 2D and 3D cultures, we carried out this present study. We compared the cell viability, the cellular morphology, the oxidative stress defensive markers, and the apoptotic and antiapoptotic signals between the 2D and 3D cultures in the T24 cell line (a balder cancer cell line). For interpretation we developed a diagrammatic model to emphasize the mass transport in part to be an important role affecting such an outcome. 

## 2. Materials and Methods

### 2.1. Chemicals and Kits

Ferulic acid (FA) was supplied by Sigma Aldrich (Saint Louis, MO, USA). The medium McCoy's 5A was provided by (GIBCO, USA), which was supplemented with 10% fetal bovine serum (FBS) (GIBCO, USA), 100 IU/mL penicillin, and 100 *μ*g/mL streptomycin (GIBCO, USA). The pH of 2D culture was unadjusted after incubation, while that of 3D cultures was controlled through CO_2_ atmosphere. The fixing solution was prepared by dissolving glutaraldehyde (2.5 g) and paraformaldehyde (2 g) in 100 mL of 0.2 M of sodium cacodylate (CaCo). The washing buffer was prepared by dissolving 7 g of sucrose in 100 mL of 0.1 M CaCO. Ferulic acid (FA) stock solution was prepared by dissolving authentic ferulic acid in DMSO to make a 2 M solution (stock solution). The required experimental solutions were prepared by diluting the stock solution with appropriate amount of medium McCoy's 5A to the experimental concentrations as indicated. Antibodies Bcl-2 (1 : 1000), Bax (1 : 1000), cleaved caspase-3 (1 : 1000), cleaved caspase-9 (1 : 1000), and *β*-actin (1 : 1000) were purchased from Bioscience Co. (United Kingdom).

### 2.2. Cells

The human urinary bladder cancer cell line, T24 (HTB-4, ATCC), was purchased from the Food Industry Research and Development Institute (Hsinchu, Taiwan, ROC). T24 cells were derived from an invasive bladder tumor of grade 3, having *p53* nonsense mutation at codon 126 (TAC to TAG) [8].

### 2.3. Cell Culture

#### 2.3.1. 2D Culture of T24 Cell Line

According to the method of [9], T24 cells at a density of 2 × 10^4^ cells/mL were seeded onto a 6-well plate in medium McCoy's 5A containing 2 mM FA. The cells were incubated at 37°C in a humidified atmosphere containing 5% CO_2_ in air for 24 h. The cultivation of T24 cells was maintained within 20 passages. These cells were further used for cultivation in RWV. 

#### 2.3.2. 3D Culture of T24 Cell Line

The T24 cells were harvested from the 2D plate culture. By following the manufacturer's instructions, the cell count was enumerated and inoculated at 2 × 10^5^ cells/mL to the 50 mL spinner vessel (Techne) of the Rotary Cell Culture System (RCCS) (Synthecon Co., Houston, TX, USA), which has been always referred to as the three-dimensional rotating-wall vessel (RWV) [10]. CultiSpher-G was prepared according to instructions and the amount used was either 2 g/L (Vero) or 1 g/L (GMK). Medium McCoy's 5A was used to fill up the entire vessel to get rid of the air. The RWV containing the medium and cells was incubated at 37°C at an agitation speed 45 rpm. The incubation was continued and the medium was replaced every 2 days together with 25 mL of sterilized FA (4.0 mM) solution to sustain the FA concentration at 2 mM. On day 3, the cells were harvested and transferred into a centrifuge tube and centrifuged at 10000 ×g for 10 min. The supernatant was decanted. The cell cluster was rinsed thrice with sterilized PBS, each time with 20 mL.

### 2.4. SEM Examination of Morphological Changes

The cells were diverged in the fixing fluid for 2 h and then centrifuged. The fixing fluid was decanted off. The residual cells were rinsed with washing buffer thrice, each time for 10 min. The rinsed cells were remained in the rinsing solution until SEM scanning. 1 *μ*L of the sample was measured from the sampling port of RCCS. Cryofixation of the suspension was finished in HPM 010 high Pressure Freezing Machine (TESCAN, USA). Graphene support films for electron microscopy (Electron Microscopy Sciences) were used. The specimens were uniformly coated with one layer gold powder using EMS 150R boast (Electron Microscopy Sciences). EM image was taken by the transmission electron microscope (DELONG TEM LVEM5) operated at an accelerating voltage 100 kV. The aperture was set at “1” with a motorized JSM-840A (Deben) to fit the TEM console. The electron gun was made of lanthanum hexaboron operated at 1500 K.

### 2.5. Cell Viability Assay

#### 2.5.1. MTT Assay for 2D Culture

According to the method described by [9], T24 cells were seeded at a density of 5 × 10^3^ cells/well onto a 24-well plate, treated with FA (DMSO vehicle, 2 and 4 mM), and incubated at 37°C in a humidified atmosphere containing 5% CO_2_ in air for 24, 48, and 72 h. To each well 0.2 mL of MTT was added and the cultivation was continued for additional 3.5 h. The MTT solution was removed by sucking off, and 0.5 mL DMSO was added to dissolve the blue formazan precipitate. After 10 min, the optical density was read at 570 nm and the cell viability was evaluated. 

#### 2.5.2. Cell Enumeration for 3D Culture

Following the manufacturer's instructions, duplicate samples of 0.5 mL were taken from the spinner. After sedimentation of the beads, 0.3 mL supernatant was withdrawn and 0.8 mL dispase (5 mg/mL in PBS) was added. Beads were completely dissolved after 30 min at 37°C. Cells were collected by centrifugation at 12000 ×g and 1.0 mL of citric acid (0.1 M) containing Triton X-100 (1%, w/v) and crystal violet (0.01%, w/v) added. Stained nuclei were counted in a hematocytometer (Percell Biolytica Application Note 115). 

### 2.6. ELISA for Serum Superoxide Dismutase and Catalase

The levels of superoxide dismutase (SOD) and catalase were measured by the SOD and catalase ELISA Kits provided by PeproTech Inc. (NJ, USA). All ELISA protocols were performed according to manufacturer's instructions. The readings were conducted with the SYSMEX K-1000 Reader, a product of San-Tong Instrument Co. (Taipei, Taiwan).

### 2.7. Western Blotting

The cells (approximately 100 mg) obtained in the above were homogenized with the homogenizer (T10 basic, The IKA Company, Germany) in 1 mL of Pro-PREP lysis buffer (pH 7.2). The homogenate was centrifuged at 12000 ×g for 20 min at 4°C, and the supernatant was collected as cell lysate sample. The cell lysate was heated at 100°C for 10 min before loading and separated on precasted 7.5% SDS-PAGE. The protein content was analyzed before loading according to the manufacturer's instructions. Aliquots of the treated lysates containing protein 50 *μ*g/*μ*L were electrotransferred onto the PVDF membrane in transfer buffer for 1 hr. The nonspecific binding to the membrane was blocked for 1 hr at room temperature with 5% nonfat milk in TBS buffer. The membranes were then incubated for 16 hr at 4°C with various primary antibodies. After extensive washing in TBS buffer, the membranes were then incubated with secondary antibody in blocking buffer containing 5% nonfat milk for 1 hr at room temperature. Membranes were then washed with TBS buffer, and the signals were visualized using the Luminescent Image Analyzer LAS-4000 (Fujifilm, Tokyo, Japan). Levels of Bcl-2, Bax, cleaved caspase-9, cleaved caspase-3, and *β*-actin were assayed, respectively, by immunoassay according to the manufacturers' instructions. *β*-actin was used as the reference protein. 

### 2.8. Statistical Analysis

Data obtained in the same group were analyzed by Student's *t*-test with computer statistical software SPSS 10.0 (SPSS, Chicago, IL, USA). ANOVA software statistical system was used with Tukey's testing to analyze the variances and significances of difference between paired means. Significance of difference was judged by a confidence level of *P* < 0.05.

## 3. Results and Discussions

### 3.1. SEM Scanning Revealed Ferulic Acid and 3D Microenvironment Induced Cell Elongation

**Figure 1:**
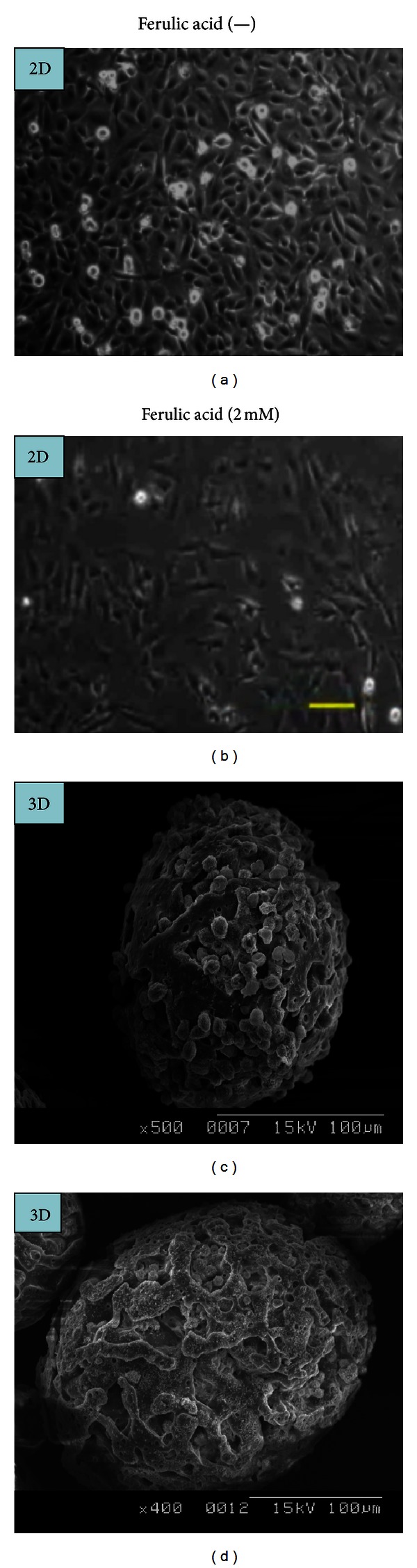
SEM scanning of the T24 cell morphology affected in the absence and presence of 2 mM ferulic acid in 2D and 3D cultures. 2D culture: control (a); 2D + ferulic acid 2 mM (b) (magnification ×500, scale bar = 0.1 mm). 3D culture: control (c) (magnification ×500); 3D + ferulic acid 2 mM (d) (magnification ×400). Cultivation time was 72 h at 37°C for 2D and 3D cultures, respectively.

In the absence of FA, T24 cells proliferated equally well despite 2D (Figure 1(a)) or 3D (Figure 1(c)) cultures. The presence of FA (2 mM) slightly elongated the cell shape, and in parallel the cell number was largely reduced (Figure 1(b)). The majority of the cells died after being cultivated for 72 h at 37°C in both 2D and 3D cultures. The dead cells on the 3D matrix appeared puffy, elongated, and not well shaped (Figure 1(d)).

### 3.2. Cell Viability Affected by Ferulic Acid and Microenvironment

**Figure 2:**
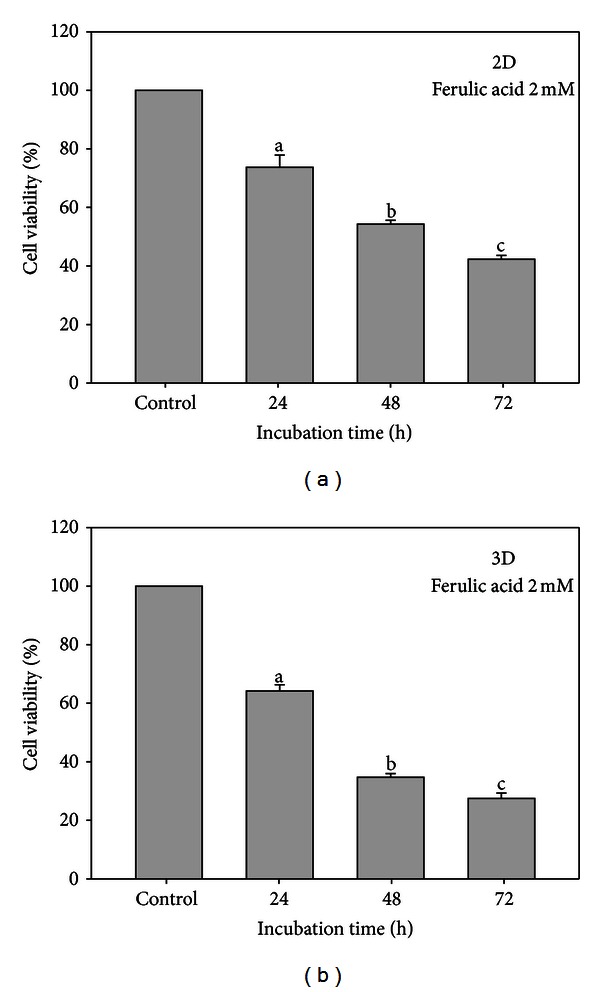
Comparison of cell viability of T24 cell lines in the presence of ferulic acid. 2D culture (a) and 3D culture (b). The concentration of ferulic acid used was 2 mM. The cultivation was conducted at 37°C for 24, 48, and 72 h, respectively. Data was expressed in mean ± SD from triplicate experiments (*P* < 0.05).

The viability of T24 cell line was seen inhibited in time-responsive manner in the presence of 2 mM FA. In 2D culture, the cell viability was reduced to 72, 53, and 36%, respectively, compared to the control (Figure 2(a)). The corresponding values reached 63, 32, and 24%, respectively (Figure 2(b)). The killing rate in 2D culture was found to be 3 × 10^5^ cells mmol^−1^·h^−1^. More severe killing rate in the 3D cultures reached 2.63 × 10^6^ cells mmol^−1^·h^−1^, giving a difference of 8.77-fold. 

Cherng et al. indicated that FA (4 mM) effectively suppressed the proliferation of J82 cells, another bladder cancer cell line, to a viability 48.79% [9]. In contrast to our data, 64% of T24 cells in the 2D and 76% in the 3D cultures were killed by ferulic acid (2 mM) after being incubated for 72 h (*P* < 0.01) (Figures 1 and 2), evidently implicating the astonishing effect caused by different microenvironments. Alternatively, different cell lines responded differently to the same phytochemical in equal strength even in the 2D culture [10]. Thus, the outcome of chemicobiological interaction depends not only on the microenvironmental factor but also on the cell genotypes. 

Rhee demonstrated nonrandom genetic and phenotypic changes in prostate epithelial cells. The occurrence of such permanent changes may be highly contact dependent and appears to be driven by specific microenvironmental factors surrounding tumor cell epithelium grown as 3D prostate organoids [11].

Why could 3D culture exhibit a higher killing rate? This part will be discussed in Section 3.6.

### 3.3. Effect of Ferulic Acid on the Superoxide Dismutase and Catalase Activities

**Figure 3:**
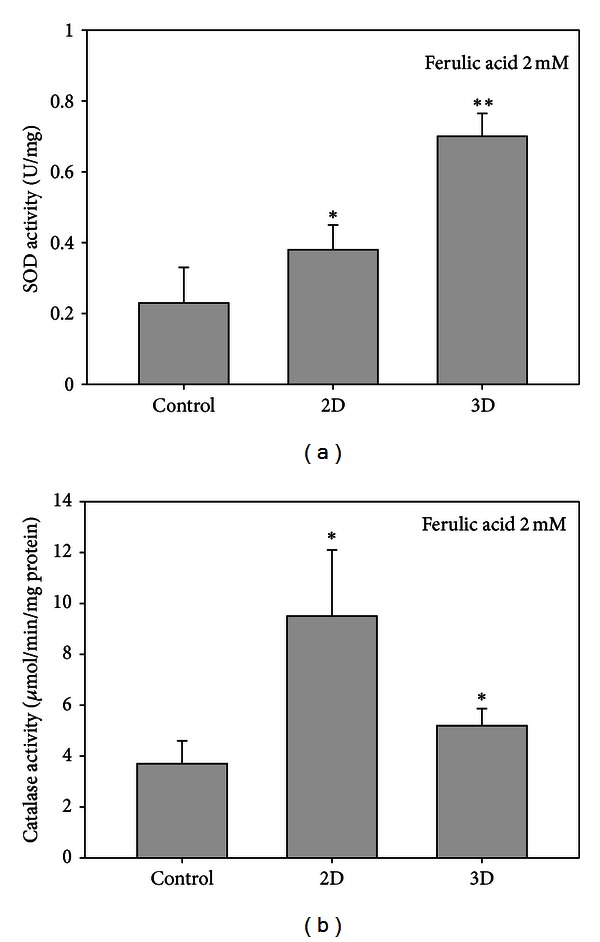
Variation of superoxide dismutase and catalase activities in T24 cell lines caused by 2D and 3D cultures. Superoxide dismutase (a) and catalase (b). Since the 2D without ferulic acid showed the lowest levels of SOD and catalase, we used the 2D without FA as the controls.

The activity of SOD was upregulated by FA in 2D and 3D cultures, increasing approximately to 1.73-fold (*P* < 0.050) and 3.18-fold (*P* < 0.001), respectively (Figure 3(a)). Conversely the catalase activity was highly induced by 2D but only slightly significant by 3D cultures. The increase reached 2.58-fold for 2D and 1.33-fold for 3D cultures (Figure 3(b)). 

Superoxide is one of the main reactive oxygen species (ROS) in the cells. Approximately 0.4–4% of all oxygen consumed during normal respiration is converted into superoxide within the mitochondrion [12], the chief source of reactive oxygen species (ROS) within the cell. Superoxide dismutases (SOD, EC1.15.1.1) are enzymes that catalyze the dismutation of superoxide into oxygen and hydrogen peroxide. Thus, they are an important antioxidant defense in nearly all cells exposed to oxygen [13]. It is simply stated that SOD outcompetes damaging reactions of superoxide, thus protecting the cell from superoxide toxicity [14]. Superoxide is known to denature enzymes, oxidize lipids, and fragment DNA. SODs catalyze the production of O_2_ and H_2_O_2_ from superoxide (•O_2_^−^), which results in less harmful reactants. When acclimating to increased levels of oxidative stress, SOD concentrations typically increase with the degree of stress conditions [15].

Catalase is one of the most potent catalysts known. The reactions it catalyses are crucial to life. Catalase catalyses conversion of hydrogen peroxide, a powerful and potentially harmful oxidizing agent, to water and molecular oxygen. Hydrogen peroxide is a harmful by-product of many normal metabolic processes: to prevent damage to cells and tissues, it must be quickly converted into other, less dangerous substances. To this end, catalase is frequently used by cells to rapidly catalyze the decomposition of hydrogen peroxide into less reactive gaseous oxygen and water molecules [16]. Catalase also uses hydrogen peroxide to oxidize toxins including phenols and alcohols [17]. Catalase is essential to protect the stability of ferrous ion-requiring enzymes both *in vitro* and *in vivo* systems [18]. Hydrogen peroxide has recently been shown to inactivate the enzyme by oxidation of crucial cysteines [19]. 

Alternatively, space microenvironment could play an important role in balancing these antioxidative enzymes. Speculatively, the synergistic interaction of SOD and catalase was effectively operating in the T24 cells and obviously there might have been much higher ROS occurring in the 2D culture, as evidenced by the highly raised catalase activity (Figure 3(b)). The major part of superoxide anions produced could have been consumed through the NO pathway by the huge amount of NO otherwise produced (not shown); hence the apparent level of SOD was highly suppressed in the 2D cultures (Figure 3(a)). Such a case was not seen in the 3D culture. In 3D culture, the T24 cells were freely rotating with the matrix and had much larger space for cell proliferation. Consequently, the ROS produced could rapidly diffuse out the cells, immediately diluted by the bulk fluid around the cells. The lower catalase activity found for the 3D culture may give a strong support to this (Figure 3(b)). 

### 3.4. Western Blot Indicated 3D Culture Showed Stronger Apoptotic Effect than 2D Culture

**Figure 4:**
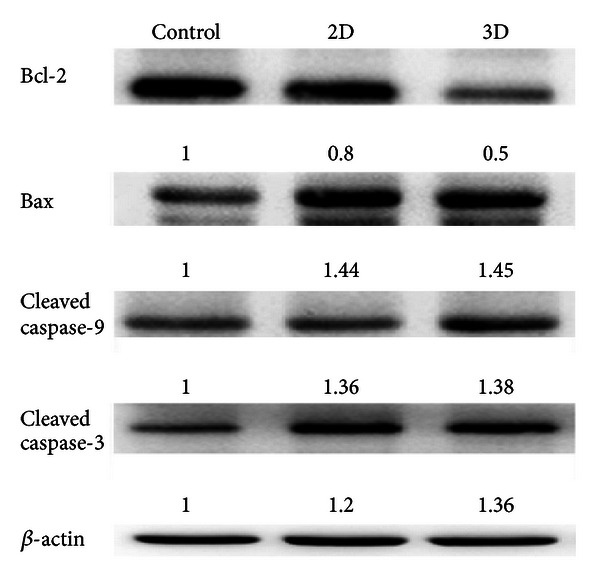
Western blot of Bcl-2, Bax, Bad, cleaved caspase-3, and cleaved caspase-9.

As seen, the antiapoptotic cytokine Bcl-2 was suppressed by 3D to 0.5-fold compared with 0.80-fold by the 2D and 1.00-fold in the control. Conversely, the proapoptotic cytokine Bax was similarly upregulated by 3D and 2D, reaching 1.45- and 1.40-fold, respectively, compared with the control, 1.00-fold. Alternatively, the cleaved caspase-9 and cleaved caspase-3 were all substantially upregulated by 3D and 2D cultures. However it is worth noting that cleaved caspase-3 was more evidently induced by 3D than 2D cultures (Figure 4). Results implicated that 3D culture exhibited higher cytotoxicity than 2D identity. 

### 3.5. Intrinsic Mitochondrial Pathway Was Involved in Apoptosis

Western blotting revealed the Bcl-2 level was more prominently downregulated by 3D (*P* < 0.05); conversely, the levels of Bax, cleaved caspase-3, and cleaved caspase-9 were all significantly upregulated in 2D and 3D cultures (*P* < 0.05), comparatively, upregulated slightly higher in 3D culture (Figure 4), an implication in the higher extent of apoptosis occurring in 3D culture (Figure 2). 

### 3.6. Difference in Mass Transport in Part Can Impart the Causality of Cell Death

**Figure 5:**
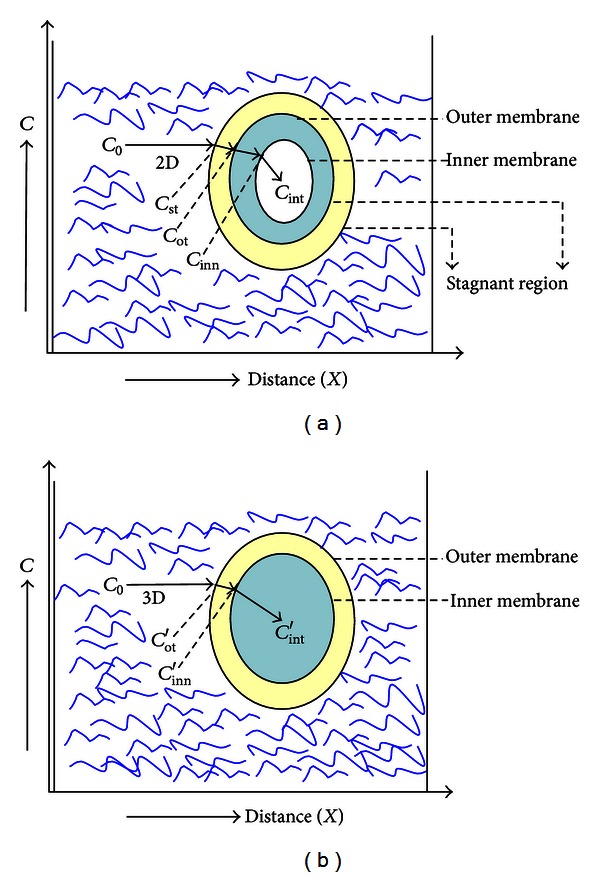
Diagrammatic model showing the difference of mass transports between the 3D (a) and 2D (b) cultures. In this model, due to the constantly free rolling of cells with the matrix in the medium for the 3D mass transport (a), the stagnant region could not be created. As contrast, the 2D mass transport (b) encounters two barriers: the stagnant region and the cell membrane. The stagnant region is a spontaneous barrier for mass transport adjacent to any membrane or catalyst surface. There would occur a concentration drop no sooner than the cells start to consume the medium.

To interpret the difference of the microenvironmental factor, we established the diagrammatic figure to show the difference of mass transport in these two cultures (Figure 5). The mass transport in 2D culture is limited by two barriers: “the stagnant region” and “the cell membrane” [20] (Figures 5(a) and 5(b)). The so-called “stagnant region” always occurs near the junction of a membrane or the surface of catalysts. Diagrammatically, the bulk concentration of ferulic acid in 2D-culture medium (*C*_o_) is first transported to the surface of stagnant region (denoted by *C*_st_, the concentration at the surface of stagnant region), dropping to *C*_ot_ (the concentration at the outer membrane) (1) and then to *C*_inn_ (the concentration at the junction of inner membrane; the subscript mem denotes “membrane”) (2), and eventually is assumed by the cells, and hence the concentration drops to *C*_int⁡_ (the intracellular concentration) (3), (4) (Figure 5(a)) (1)–(4):

(1) $$\begin{array}{c} C_{\text{ot}}=C_{\text{o}}-\int_{t1}^{t2}{\left(\frac{\delta C}{\delta x}\right)}_{\text{st}}{\left(\frac{\delta x}{\delta t}\right)}_{\text{st}}, \end{array}$$

(2) $$\begin{array}{c} C_{\text{inn}}=\left(\frac{C_{\text{ot}}}{\delta x}\right){\left(\frac{\delta x}{\delta t}\right)}_{\text{mem}}, \end{array}$$

(3) $$\begin{array}{c} {\left(\frac{\delta C}{\delta t}\right)}_{\mathrm{int}}=k_{2\text{D}}\left[C\right]. \end{array}$$

Integration of (3) yields

(4) $$\begin{array}{c} \int_{C_{\text{inn}}}^{C_{\mathrm{int}}}\frac{dC}{C}=k_{2\text{D}}\int_{t1}^{t2}dt, \end{array}$$

where *K*_2D_ is the consuming rate coefficient in 2D culture. 

In contrast, due to the constantly free rolling of the cells with matrix in the medium, the stagnant region may disappear or be neglected in the 3D culture, where the bulk concentration (*C*_o_) directly drops from the concentration at the outer membrane (*C*_ot_′) to the concentration at the inner membrane (*C*_inn_′) (5) and then degraded to the intracellular concentration (*C*_int⁡_) (6), (7) (Figure 5(b)) (5)–(7):

(5) $$\begin{array}{c} C_{\text{inn}}=\left(\frac{C_{\text{ot}}^{\prime }}{\delta x}\right){\left(\frac{\delta x}{\delta t}\right)}_{\text{mem}}, \end{array}$$

(6) $$\begin{array}{c} {\left(\frac{\delta C}{\delta t}\right)}_{\mathrm{int}}=k_{3\text{D}}\left[C\right]. \end{array}$$

Integration of (6) yields

(7) $$\begin{array}{c} \int_{C_{\text{inn}}^{\prime }}^{C_{\mathrm{int}}^{\prime }}\frac{dC}{C}=k_{3\text{D}}\int_{t1}^{t2}dt, \end{array}$$

where *K*_3D_ is the consuming rate coefficient in 3D culture. 

Obviously without the presence of stagnant region in 3D culture, the mass transport will be less hindered. Hence, the cells in 3D culture would frequently “feel” or encounter higher toxicant concentration than the 2D culture. Moreover, the elongation of cells in 3D culture would provide a larger surface area for mass transport; as a consequence, the transport would fulfill the conditions *C*_inn_′ ≫ *C*_inn_ and *C*_int⁡_′ ≫ *C*_int⁡_, and the 3D cultures could be affected by ferulic acid at higher concentration. Thus although 3D culture may have many advantages: (i) largely reducing shear and turbulence generated by conventional stirred bioreactors, (ii) minimizing mechanical cell damage, and (iii) continuously free falling, promoting the assembly of 3D cellular aggregates which allow a microenvironment for more efficient cell-to-cell interactions and exchange of growth factors [7], we suggest that the 3D RWV culture cannot be as real as the *in vivo *3D growth. 

To summarize, experiment with the T24 cell lines-ferulic acid (2 mM) model reveled that FA showed different cytotoxicity on T24 cell line in the 2D and 3D culture systems. The cell shape was more elongated in the 3D culture. The SOD activity was higher, and conversely, the catalase activity was lower in the 3D culture. The antiapoptotic signal Bcl-2 was downregulated, while all the apoptotic signals Bax, cleaved caspase-3, and cleaved caspase-9 were upregulated by FA, despite in the 2D or the 3D cultures. The overall apoptotic rate was higher for the 3D culture. To extend, more complicated pharmacokinetic and pharmacodynamic events could be expected in the *in vivo* 3D tissues. 

## 4. Conclusions

Apparently, 3D culture has shown more powerful cytotoxicity than the 2D analogue. In cancer therapy, we encourage that the outcome of 2D culture must be corrected for the results to be applied to the *in vivo* cancer treatment.

## Supplementary Material

Supplemental Figure: A scheme for producing RWV-1, -2, and -3 cells from the 3D prostate organoids cultured under simulated microgravity conditions with either microcarrier beads alone (RWV-1), or with prostate (RWV-2), or bone (RWV-3) fibroblasts (depicted from Rhee et al., 2001 [11]).

## References

[1] Ou S, Kwok KC (2004). Ferulic acid: pharmaceutical functions, preparation and applications in foods. *Journal of the Science of Food and Agriculture*.

[2] Ross JA, Kasum CM (2002). Dietary flavonoids: bioavailability, metabolic effects, and safety. *Annual Review of Nutrition*.

[3] Hsieh CL, Peng CC, Cheng YM (2010). Quercetin and ferulic acid aggravate renal carcinoma in long-term diabetic victims. *Journal of Agricultural and Food Chemistry*.

[4] Peng CC, Hsieh CL, Wang HE, Chung JY, Chen KC, Peng RY (2012). Ferulic acid is nephrodamaging while gallic acid is renal protective in long term treatment of chronic kidney disease. *Clinical Nutrition*.

[5] Wang R, Xu J, Juliette L (2005). Three-dimensional co-culture models to study prostate cancer growth, progression, and metastasis to bone. *Seminars in Cancer Biology*.

[6] Sung SY, Hsieh CL, Wu D, Chung LWK, Johnstone PAS (2007). Tumor microenvironment promotes cancer progression, metastasis, and therapeutic resistance. *Current Problems in Cancer*.

[7] Synthecon Rotary cell culture system (RCCS 8DQ) from Synthecon, Biocompare. http://www.synthecon.com/.

[8] Bubenik J, Baresova M, Viklicky V (1973). Established cell line of urinary bladder carcinoma (T24) containing tumour specific antigen. *International Journal of Cancer*.

[9] Cherng JM, Shieh DE, Chiang W, Chang MY, Chiang LC (2007). Chemopreventive effects of minor dietary constituents in common foods on human cancer cells. *Bioscience, Biotechnology and Biochemistry*.

[10] Peng CC, Chen KC, Peng RY, Chyau CC, Su CH, Hsieh-Li HM (2007). Antrodia camphorata extract induces replicative senescence in superficial TCC, and inhibits the absolute migration capability in invasive bladder carcinoma cells. *Journal of Ethnopharmacology*.

[11] Rhee HW, Zhau HE, Pathak S (2001). Permanent phenotypic and genotypic changes of prostate cancer cells cultured in a three-dimensional rotating-wall vessel. *In Vitro Cellular & Developmental Biology*.

[12] Hansford RG, Hogue BA, Mildaziene V (1997). Dependence of H_2_O_2_ formation by rat heart mitochondria on substrate availability and donor age. *Journal of Bioenergetics and Biomembranes*.

[13] Li Y, Huang TT, Carlson EJ (1995). Dilated cardiomyopathy and neonatal lethality in mutant mice lacking manganese superoxide dismutase. *Nature Genetics*.

[14] Melov S, Doctrow SR, Schneider JA (2001). Lifespan extension and rescue of spongiform encephalopathy in superoxide dismutase 2 nullizygous mice treated with superoxide dismutase-catalase mimetics. *Journal of Neuroscience*.

[15] Alscher RG, Erturk N, Heath LS (2002). Role of superoxide dismutases (SODs) in controlling oxidative stress in plants. *Journal of Experimental Botany*.

[16] Gaetani GF, Ferraris AM, Rolfo M, Mangerini R, Arena S, Kirkman HN (1996). Predominant role of catalase in the disposal of hydrogen peroxide within human erythrocytes. *Blood*.

[17] Boon EM, Downs A, Marcey D Proposed mechanism of catalase. http://biology.kenyon.edu/BMB/Chime/catalase/frames/cattx.htm#Proposed%20Mechanism%20of%20Catalase.

[18] Werner ER, Werner-Felmayer G (2007). Substrate and cofactor requirements of indoleamine 2,3-dioxygenase in interferon-gamma-treated cells: utilization of oxygen rather than superoxide. *Current Drug Metabolism*.

[19] Poljak A, Grant R, Austin CJD (2006). Inhibition of indoleamine 2,3 dioxygenase activity by H_2_O_2_. *Archives of Biochemistry and Biophysics*.

[20] Bailey JE, Ollis DF (198). *Biochemical Engineering Fundamentals International Edition*.

