# Supplementary material for: Cytotoxicity of Ferulic Acid on T24 Cell Line Differentiated by Different Microenvironments
Source: Biomed Res Int. 2013 May 8;2013:579859. doi: 10.1155/2013/579859 (PMC3662181; doi:10.1155/2013/579859)
Supplement: Supplementary file 1 — Supplemental Figure: A scheme for producing RWV-1, -2, and -3 cells from the 3D prostate organoids cultured under simulated microgravity conditions with either microcarrier beads alone (RWV-1), or with prostate (RWV-2), or bone (RWV-3) fibroblasts (depicted from Rhee et al., 2001 [11]). [file 579859.f1.pdf]

Supplement

Fig. S1.

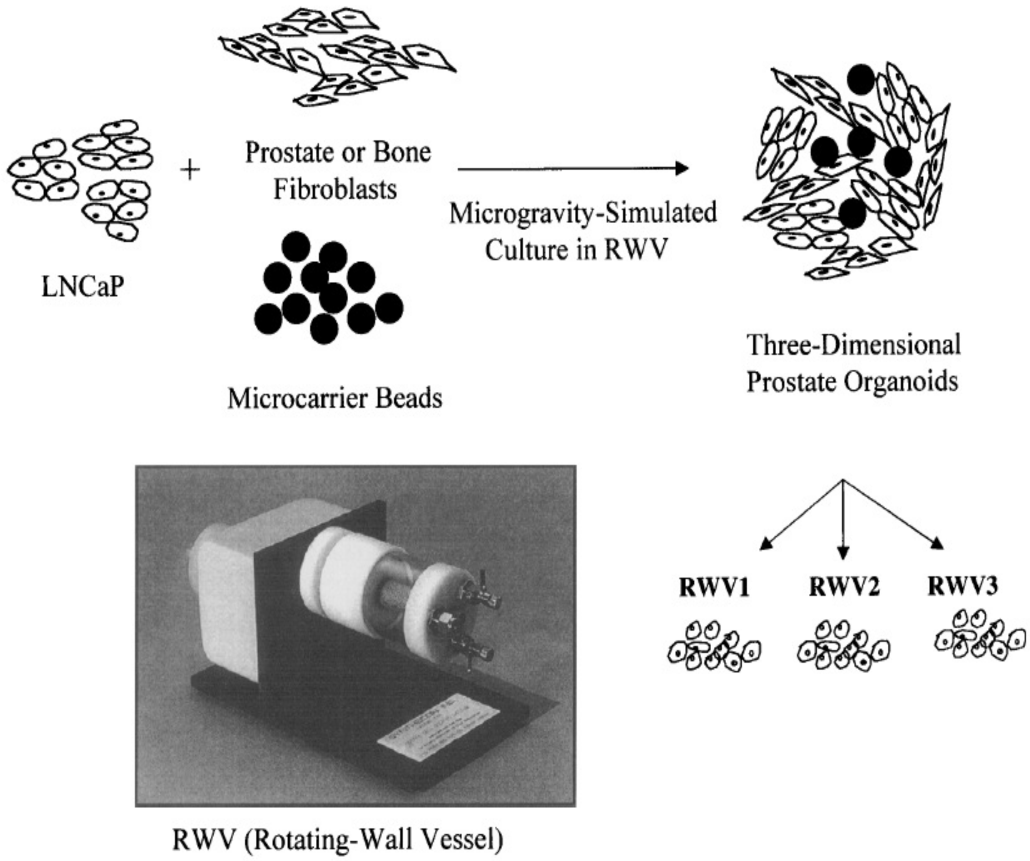

The NASA-engineered rotating wall vessel (RWV) tissue culture bioreactor system, manufactured by Synthecon (Houston, TX), addresses this need [7] (Fig. S1, Supplement).
